# Posterior Reversible Encephalopathy Syndrome Secondary to Varicella Encephalitis

**DOI:** 10.7759/cureus.12484

**Published:** 2021-01-04

**Authors:** Huzaifa Dawood, Saad Nasir, Mushtaq Ahmed, Claire O'Brien, Mustafa Dawood

**Affiliations:** 1 Cardiology, Naas General Hospital, Tralee, IRL; 2 Internal Medicine, United Medical and Dental College, Creek General Hospital, Karachi, PAK; 3 Pediatrics, Our Lady of Lourdes Hospital, Drogheda, IRL; 4 Medicine, University Hospital Kerry, Tralee, IRL; 5 Nephrology, Emory University School of Medicine, Atlanta, USA

**Keywords:** varicella encephalitis, posterior reversible encephalopathy syndrome, infections

## Abstract

Posterior reversible encephalopathy syndrome (PRES) is a rapidly progressive neurologic condition presenting with typical symptoms including headache, nausea, vomiting, altered mental status, and visual defects. Neuroimaging profile, particularly magnetic resonance imaging (MRI), is the most important tool for diagnosis. The most commonly reported etiological factors include hypertensive emergency and renal disease. We describe a 67-year-old lady who developed clinical and radiological characteristics of PRES secondary to Varicella encephalitis.

## Introduction

Posterior reversible encephalopathy syndrome (PRES) is an acute neurological syndrome characterized by symmetric bilateral vasogenic edema in the parietal and occipital regions of the cortex, evident on magnetic resonance imaging (MRI) [1]. Typical symptoms include headache, nausea, vomiting, altered mental status, visual defects, and motor abnormalities. It is associated with multiple etiologic factors, but the infectious cause predominantly includes gram-positive organisms [2]. Of the viruses, human immunodeficiency virus (HIV) and influenza A encephalitis are a known cause of PRES [3,4]. However, clinical and neuroimaging consistent with PRES is not reported with Varicella encephalitis.

Here, we present a case of a lady, admitted after a seizure episode and altered sensorium with MRI findings suggestive of PRES secondary to Varicella encephalitis.

## Case presentation

A 67-year-old lady with a background history of type 1 diabetes mellitus presented this in the emergency department of a tertiary care hospital with generalized tonic-clonic seizures involving all four limbs, lasting for about two minutes, with no urinary or fecal incontinence and having postictal confusion. Blood sugar measured on arrival was normal.

On physical examination, her blood pressure was 130/65 mmHg, pulse 82/min, respiratory rate 11/min, and temperature was 34.1°C. Neurological examination showed Glasgow coma scale (GCS) was 13/15 (eye 4, verbal 4, and motor 5), cranial nerves were grossly intact, and she was moving all four limbs, but not following commands. CT scan of the brain was normal. Her laboratory investigations including metabolic profile were normal.

During the second day of admission, the temperature spiked to 38.3°C and her GCS dropped to 7/15 (eye 2, verbal 2, motor 3), although she remained hemodynamically stable, but we shifted her to the ICU. We then started her on intravenous ceftriaxone and intravenous acyclovir for suspected meningoencephalitis.

**Figure 1:**
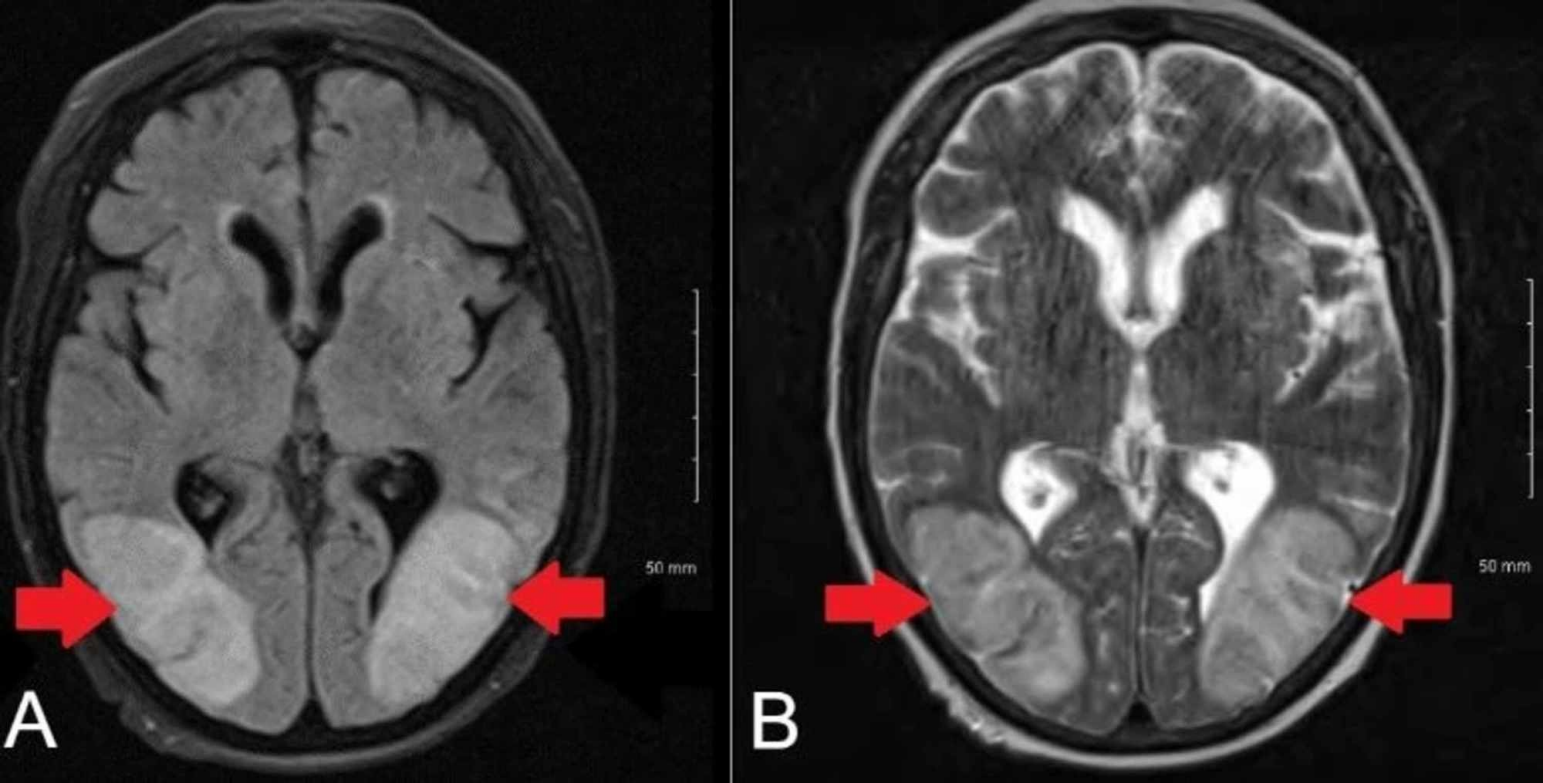
Magnetic resonance imaging axial view of the brain. (A) T1-weighted image revealing expansile region of lateral aspect of both occipital lobes. (B) Large relatively confluent expansile regions of T2 fluid-attenuated inversion recover (FLAIR) hyperintensity in the lateral aspects of both occipital lobes.

MRI revealed largely relative confluent expansile regions of T2 fluid-attenuated inversion recovery (FLAIR) hyperintensity and T1 hypointensity in the bilateral parietal lobes, right posterior frontal lobe, lateral aspect of both occipital lobes, and left posterior temporal lobe (Figure 1). Diffusion-weighted imaging (DWI) showed large facilitated diffusion in the involved subcortical and deep white matter. Based on these neuroimaging findings, we diagnosed the patient with PRES. Cerebrospinal fluid (CSF) analysis was in the reference ranges. No organisms were observed on CSF Gram stain and culture. However, Varicella-Zoster Virus (VZV) DNA was detected in CSF by polymerase chain reaction (PCR). The patient’s clinical condition improved on day 3 of admission. We continued intravenous acyclovir therapy for 21 days, and the patient was successfully discharged home.

## Discussion

PRES, first described by Hinchey et al., is a reversible neurological syndrome presenting with headache, altered mental status, and loss of vision with classical neuroimaging findings indicating posterior encephalopathy. The VZV is associated with various neurological manifestations including ischemic or hemorrhagic stroke and transient ischemic attacks. Aside from vasculopathy, VZV can infect meninges, brain parenchyma, and nerve roots to cause meningitis, encephalitis, and meningo-radiculitis [5]. 

A trigger is usually identified in the development of PRES. It most commonly manifests in the hypertensive emergency secondary to eclampsia or acute kidney injury. Other etiological factors include chronic illnesses, autoimmune conditions, immunosuppressive medications such as tacrolimus, cyclosporine, or chemotherapeutic agents [6]. PRES following an infection is believed to occur because of toxin-mediated endothelial cell dysfunction, which results in increased vascular permeability and ultimately vasogenic edema; similar to the pathogenesis involved in both hypertension and immunosuppression related [7]. The diagnosis is established based on the presence of risk factors and characteristic imaging findings. Of which, the most commonly observed pattern includes vasogenic edema evident in the parieto-occipital region [8].

Our patient presented with clinical features of PRES, but during her hospital stay, she developed a fever and her progressively worsening neurological status leads us to consider other possible causes. We later diagnosed our patient with PRES secondary to Varicella encephalitis based on the detection of VZV DNA in CSF using PCR. Cao et al. reported a case of Varicella encephalitis presenting as a lateral medullary syndrome [9]. However, to the best of our knowledge, Varicella encephalitis with clinical and radiological findings consistent with PRES has never been described in the literature before.

Seibert et al. conducted a retrospective study to determine the factors associated with poor outcomes in patients with PRES. A review of their findings reveals that the most common etiology associated with in-hospital mortality included sepsis and chemotherapy. While, higher age, female gender, presence of subarachnoid hemorrhage, increased C-reactive protein levels and altered mental status at onset were the other factors [10]. Management focuses on prompt initiation of supportive care and removal of inciting factors [8].

While the role of VZV in PRES is uncertain, simultaneous presentation of both conditions reinforces the observation that PRES can be triggered by VZV. Early administration of acyclovir in our case resulted in rapid clinical improvement and physicians should be aware of this possibility.

## Conclusions

Although recognition of viral diseases as triggers of PRES exists in the literature, we report a unique association of PRES with VZV. We further recommend the conduction of epidemiological studies to better understand the pathophysiological mechanisms involved, as early identification leads to better patient outcomes.
